# Peer mentorship and professional identity formation: an ecological systems perspective

**DOI:** 10.1186/s12909-024-05992-0

**Published:** 2024-09-15

**Authors:** Lalit Kumar Radha Krishna, Nur Amira Binte Abdul Hamid, Gillian Li Gek Phua, Stephen Mason, Ruaraidh Hill, Crystal Lim, Simon Yew Kuang Ong, Eng Koon Ong, Halah Ibrahim

**Affiliations:** 1 Yong Loo Lin School of Medicine, National University of Singapore, NUHS Tower Block, Level 111E Kent Ridge Road, Singapore, 119228 Singapore; 2 Division of Cancer Education, National Cancer Centre Singapore, 30 Hospital Boulevard, Singapore, 168583 Singapore; 3 Division of Supportive & Palliative Care, National Cancer Centre Singapore, 30 Hospital Boulevard, Singapore, 168583 Singapore; 4 Duke-NUS Medical School, National University of Singapore, 8 College Road, Singapore, 169857 Singapore; 5 Centre for Biomedical Ethics, National University of Singapore, Block MD11, 10 Medical Drive, Singapore, 117597 Singapore; 6 End of Life Care Centre, Palliative Care Institute Liverpool, University of Liverpool, Academic Palliative &200 London Road, Liverpool, L3 9TA UK; 7 PalC, The Palliative Care Centre for Excellence in Research and Education, Singapore PalC c/o Dover Park Hospice, 10 Jalan Tan Tock Seng, Singapore, 308436 Singapore; 8 Health Data Science, University of Liverpool, Whelan Building, The Quadrangle, Brownlow Hill, Liverpool, L69 3GB UK; 9 Duke-NUS Medical School, Lien Centre for Palliative Care, National University of Singapore, 8 College Road, Singapore, 169857 Singapore; 10 Medical Social Services, Singapore General Hospital, Outram Road, Singapore, 169608 Singapore; 11 Division of Medical Oncology, National Cancer Centre Singapore, 30 Hospital Boulevard, Singapore, 168583 Singapore; 12 Assisi Hospice, 832 Thomson Road, Singapore, 574627 Singapore; 13 Khalifa University College of Medicine and Health Sciences, Abu Dhabi, United Arab Emirates

**Keywords:** Professional identity formation, Mentoring, Community of practice, Professionalism, Personhood, Research, Palliative medicine

## Abstract

**Background:**

Mentoring can help shape how medical students think, feel, and act as physicians. Yet, the mechanism in which it influences this process of professional identity formation (PIF) remains poorly understood. Through the lens of the ecological systems theory, this study explores the interconnected and dynamic system of mentoring relationships and resources that support professional development and growth within the Palliative Medicine Initiative (PMI), a structured research peer mentoring program.

**Methods:**

A secondary analysis of transcripts of semi-structured interviews with peer mentors and mentees and a review of their mentoring diaries was conducted to explore the impact of participation in a longitudinal peer mentoring program on both mentees and peer mentors on their personal and professional development through the lens of the mentoring ecosystem model. The Systematic Evidence-Based Approach was adapted to analyze the data via content and thematic analysis.

**Results:**

Eighteen mentees and peer mentors participated and described a supportive community of practice within the research program, with discrete micro-, meso-, and macro-environments that are dynamic, reflexive, and interconnected to form a mentoring ecosystem. Within this ecosystem, reflection is fostered, and identity work is done—ultimately shaping and refining self-concepts of personhood and identity.

**Conclusion:**

This study underscores the nuances and complexities of mentorship and supports the role of the mentoring ecosystem in PIF. A deeper understanding of the multiple factors that converge to facilitate the professional development of mentees can help educators develop and implement structured peer mentorship programs that better support reflective practice and identity work.

## Introduction

Medical education employs mentoring to foster the development of altruistic, ethical, humanistic, and accountable clinicians through Professional Identity Formation (PIF), or how medical students learn to think, feel, and act as physicians [1–3]. However, there is a limited understanding of mentoring relationships and the dynamics between mentors, mentees, and the host organizations. The impact of contextual, sociocultural, and programmatic influences on PIF is also often unaddressed [4–8], hampering the effective use of mentoring in medical education [4–6].

The ecological systems model provides a framework for understanding the multiple factors that directly or indirectly influence behavior and relationships between individuals and between individuals and organizations [9]. It asserts that a phenomenon such as PIF within the PMI results from a convergence of factors spanning interrelated levels of influence, namely the microsystem (individual), mesosystem (group), exosystem (organizational), macrosystem (cultural and societal), and chronosystem (impact of time and life transitions) [9–12].

Through the ecological systems theory [13], we explore the interconnected and dynamic system of mentoring relationships and resources that support the mentoring environment and PIF within the Palliative Medicine Initiative (PMI), a structured research peer-mentoring program [3, 14, 15]. This study aims to investigate the mentees’ progress in the program and their development of professional identity through the lens of the mentoring ecosystem [3, 14, 15].

### The Palliative Medicine Initiative (PMI)

In Singapore, undergraduate medical education typically involves a five-year program leading to a Bachelor of Medicine and Bachelor of Surgery (MBBS) degree. Postgraduate medical education is structured, competency-based training in various medical specialties following a United States residency training model [16].

The PMI is a voluntary research mentoring program based at the National Cancer Centre Singapore (NCCS) that includes medical students and residents from Duke-NUS Medical School, National University of Singapore’s (NUS) Yong Loo Lin School of Medicine (YLLSoM), and the Lee Kong Chian Medical School. Most research projects focus on palliative care, professionalism and PIF, well-being, and medical ethics. The PMI uses a peer mentoring model, which involves collaboration between trainees of similar training stages and experiences facilitated by a senior mentor. Mentees who successfully complete at least one mentored research project to publication learn how to mentor, assess, and provide feedback to others as peer mentors [11]. The PMI’s research mentoring program is designed on best practices derived from prior systematic reviews on mentoring relationships [5, 17], and studies of the mentoring environment [18], mentor training [19, 20], and ethical issues in mentoring [21–23]. The PMI has supported over 100 mentee-led publications in peer-reviewed journals over the past 10 years [4, 24]. This success has been attributed to the PMI’s mentoring structure and culture that nurture enduring and personalized mentoring relationships [5, 17, 25].

The PMI has several key design elements [14, 15]. First, it is a structured program with a formal curriculum overseen by the Divisions of Supportive and Palliative Care and Cancer Education at NCCS (host organization). The host organization ensures a learning environment with a consistent mentoring approach, clearly delineated expectations and codes of practice, and competency-based assessments [4–6]. Next, the PMI is designed around structured research mentoring stages: mentee initiation, matching, initial meetings, data gathering, data analysis, manuscript writing, submission, and post-submission [26]. Each stage is characterized by clear competencies to be achieved and objectives to be met. Progress through the stages creates a mentoring trajectory. Further, the program employs a blend of mentoring, coaching, career guidance, and role modeling to support mentors, peer mentors, and mentees. Finally, peer mentors and mentees complete mentoring diaries that map their development and encourage reflective practice [27]. The mentoring diaries are reviewed by PMI faculty, who assess the quality of mentoring interactions and offer advice, mentoring support, and counselling support as needed [28].

### Mentoring ecosystem

The PMI encompasses a mentoring environment comprised of formal, informal, and hidden (contextual, sociocultural, ethical, and professional) influences. Together with the mentoring structure, these comprise the mentoring ecosystem [29]. The mentoring ecosystem impacts, and is impacted by, the progress of individual stakeholders. Each stakeholder operates within a discrete micro-environment [3]. Each micro-environment is shaped by five aspects of each stakeholder: 1) beliefs and principles (belief system); 2) motivations, attitudes, abilities, and experience (narratives) [30–35]; 3) clinical, academic, cultural, organizational, and societal spheres (contextual considerations) [17]; 4) developing skills, knowledge, motivations, levels of engagement, and evolving goals (developing competencies); and 5) personal development [18].

As personal micro-environments become embedded in the mentoring ecosystem, they progress to a more central position in the program as they interact with the program’s culture and structure and with other micro-environments [36–41]. Interactions between individual micro-environments create the meso-environment [42]. Within the meso-environment, each stakeholder’s evolving belief systems, contextual considerations, developing competencies, and the nature and dynamics of pre-existing relationships with one another impact other surrounding micro-environments. The program structure includes codes of practice and boundaries to prevent breaches of professional standards.

The mentoring culture and structure create the macro-environment, which helps align “identities in a manner that is congruent with the regime of competence within that… institution” [43]*.* This process of integrating the PMI’s values, beliefs, and principles and nurturing mentees to become peer mentors and peer mentors to become mentors—the socialization process—is critical to PIF [36]. In some cases, it involves “crossing a threshold leading to a change in knowing, doing, being, and future learning possibilities” [44]. Identity work, the active process and adaptations made to develop and shape professional identity, is guided by mentoring support and feedback. Pratt et al. [45] suggest that there are two forms of identity work. The first is an inexperienced mentee adopting a former identity to ‘splint’ and protect their vulnerable, developing professional identity. The second is a more experienced mentee calling upon their perception of an ideal physician to ‘permanently patch up holes’ in their existing identity (patching)*.*

## Methodology

This study is a secondary analysis of narratives from mentoring diaries and transcripts from semi-structured interviews conducted in 2021 to explore mentoring experiences within the PMI. In this study, we seek to provide deeper insights into contextual and programmatic influences on PIF to answer our research question: does the mentoring ecosystem model explain how PIF evolves within the PMI?

We adopt Krishna’s Systematic Evidence-Based Approach (SEBA) methodology to guide our efforts. SEBA enhances the rigor and transparency of research by combining systematic review techniques with qualitative synthesis [7, 14, 15, 25, 26]. The key components include a systematic literature review, qualitative data collection through semi-structured interviews, thematic analysis, and integrating the qualitative findings with existing literature [7, 14, 15, 25, 26]. This approach allows us to explore the contextual and programmatic influences on PIF within the PMI and provides a structured framework to support our findings.

The series of semi-structured interviews with individual mentees and peer mentors conducted in 2021 were triangulated against mentoring diaries and recently published accounts of mentee and peer mentor experiences in the PMI [3, 4, 26, 46]. Ethics approval was obtained from the SingHealth Combined Institutional Review Board (CIRB Ref 2020/3056).

### Systematic Evidence-Based Approach (*SEBA*)

An expert team comprised of a medical librarian, local educational experts and clinicians, and PMI alumni oversaw each aspect of the research process to ensure consistency, reproducibility, and transparency. Semi-structured interview questions were based on published accounts of mentoring experiences in the PMI [4, 46] and a recent review of mentoring programs, practices, and assessments [19]. The interview guide was also influenced by theories of communities of practice [31, 42] and PIF [8, 36, 47–51].

### Data collection

We conducted a purposive sampling of mentees and peer mentors actively involved in the PMI who had successfully completed at least one project to publication. Email invitations containing a participant information sheet and consent forms were sent to eligible participants. The invitations emphasized participant anonymity and the right to withdraw from the study at any point without prejudice. Upon the receipt of signed consent forms, semi-structured interviews were arranged with individual mentees and peer mentors. The interviews were conducted by two non-clinician researchers who had no dependent relationship with the participants and were briefed on the study aims. The interviews took approximately 30 to 45 min each and were conducted over the Zoom video conferencing platform between February and May 2021 in quiet offices to ensure privacy and facilitate in-depth exploration of personal beliefs and experiences. Verbal consent was obtained before interviews were audiotaped. The audio recordings were transcribed verbatim using NVivo 12 software [52] and the transcripts were anonymized. Mentees and peer mentors who consented to have their mentoring diaries analyzed had their entries anonymized by independent research team members not involved in the PMI or the semi-structured interviews. Data collection and analysis were conducted concurrently and led to iterative adjustments to the interview guide. Participant recruitment continued until no new themes emerged.

### Data analysis

From August through September 2023, the de-identified transcripts and diaries were reviewed and coded by two independent teams. Each team consisted of 3 mentees and was led by a peer mentor. A senior clinician supervised the data analysis. Inductive and deductive analysis were used concurrently. Using Braun and Clarke [53] qualitative data analysis methodology, one team engaged in iterative and cyclic constant comparison analysis. Any disagreements were negotiated through in-depth conversations. Through consensus, themes were identified and then applied to all transcripts. A codebook was maintained to enhance the reproducibility and trustworthiness of the data. The second group employed Hsieh and Shannon’s [54] approach to directed content analysis. Using Krishna et al.’s [3] study and Teo et al.’s [29] review, a priori coding categories were identified and applied to the transcripts to help confirm and expand the ecological systems theory to the mentoring setting [13]. The reviewers within each team achieved consensus on their analyses through discussion and negotiation before comparing with the other team. Conducting both thematic and content analyses allowed us to omit calculating Cohen’s Kappa to gauge the degree of consensus between different researchers [55].

Next, the overlapping themes and categories from each set of transcripts were combined to construct overarching themes/categories [56–58]. This process was repeated for the peer mentor and mentee mentoring diaries. Finally, the themes/categories derived from the mentoring diaries and interviews were compared, creating the domains that framed the discussion.

### Team reflexivity

As both researchers and participants in the PMI, we acknowledge our dual roles in the study. Our insider status provided valuable insights into the context and nuances of the mentorship program but also risked introducing bias. We implemented several strategies to mitigate this risk, including regular team discussions to challenge assumptions and interpretations and proactively distinguish between our personal experiences and the experiences reported by study participants. We also engaged clinicians and educators external to the PMI as co-researchers and co-authors to help leverage our insider knowledge while maintaining critical distance.

## Results

Of 18 peer mentors and 10 mentees eligible to participate in the study, twelve peer mentors and seven mentees were recruited for the semi-structured interviews. Tables 1 and 2 depict participant demographics and their experience within the PMI.

**Table 1:** Demographics of Peer Mentor (NP) Interview Participants

Peer-Mentor (NP)	Student Year	No. of projects undertaken	Duration involved in PMI (years)
NP1	PGY3	3	3
NP2	PGY1	9	2
NP3	PGY1	5	1
NP4	M4	4	1
NP5	M4	4	2
NP6	M4	6	3
NP7	M5	6	1
NP8	M5	2	1
NP9	M4	2	2
NP10	M2	5	2
NP11	M4	2	1
NP12	M4	2	1

PGY indicates medical resident and year of training

M indicates medical student and year of education

**Table 2:** Demographics of Mentee (M) Interview Participants

Mentee	Demographic	No. of projects undertaken	Duration involved (years)	Became NPs
M3	Medical student- not stated	3	(Since M1)	Soon to be
M4	M2	1	1	No
M5	M2	3–4	1	No
M6	M4	1	1	No
M8	M2	1	1	No
M10	Medical student- not stated	1	(Since M1)	No
M12	M3	4	< 1	No

M indicates medical student and year of education

### Domain 1. mentoring ecosystem

Features of a community of practice were reported [59]. M4 described the development of a social network of individuals who shared and developed overlapping knowledge bases, values, and experiences:

*“At first, I was rather stressed writing the paper. But then, as you communicate with the other mentees that were on the same stage with you, you realize that everybody is equally stressed and equally lost. And that kind of gave some comfort because we were stressed together. And then we would freak out together. So, it was a bit like a community that you could find comfort in, even though everybody was lost together. But we could also find our way out and explore and work things out together. So, it was a very supportive system in the sense that although it might be stressful, we knew that we were not alone.” M4*

This domain further contains the subdomains of micro-, meso-, and macro-environments, consistent with the mentoring ecosystem.

### Sub-domain 1. Micro-environment

Mentees and peer mentors revealed the internal elements shaping their micro-environments. M1’s vignette reflected on how individual characteristics and beliefs could affect the micro-environment:

*“Initially, I felt it was very hard to say no. So even if I wasn’t confident in the project or keen, I would still do it anyway because I...am a people pleaser… I did not want to disappoint my mentor. So initially, it was more of like whatever my mentor wanted me to do, I’d just say yes to it. Even though I didn’t know what to do [or] feel like doing it…” M1*

Internal motivations included the mentees’ desire to gain opportunities and refine their skills in order to advance their research ambitions and enhance their CVs:

*“I wanted to learn how to properly do a research paper, learn the relevant skills.” M10*

*“We all wanted to take up every single thing that came our way. So, for me then, it was really maximizing all the opportunities that came my way and trying to do as much as I could.” M4*

At times, participants expressed the chasm between the expectations of their identity as a peer mentor and their personal dispositions:

*“So, because of who I am and my character, I tend to [be] really lighthearted... But when it comes to being a near-peer, ... because I’m so lighthearted…even though I’m leading a project, I do not see myself as a leader, as someone who tells people what to do… That affected my mentorship or my ability to lead.” NP1*

Moreover, changes in motivations, competencies, and maturing mentoring relationships were influenced by changes in contextual and personal circumstances and evolved over time:

*“At first, I had a lot of doubts that maybe this wasn’t for me, because I really didn’t know what was happening… but after that, I decided to give it another try, to take on a few more papers. Throughout the process, I realized that actually I do understand if I put in the effort to seek clarifications earlier and also communicate more with the seniors to find out more on what’s happening and what is required of me.” M4*

*“I guess at the start when you’re new, you try to learn and pick up all these skills you need to write a paper. But now, your goals change a little because you actually want to write something meaningful—write something that will benefit, or think will benefit, the community in the future.’ M10*

### Sub-domain 2. Meso-environment

Mentees and peer mentors acknowledged the influence of interactions with other mentees, peer mentors, and other stakeholders in nurturing their micro-environments and in forming meso-environments. The formation of these meso-environments built confidence and relationships, guided personalized support, and changed thinking and conduct, ultimately informing their professional identity.

Mentees and peer mentors believed that their mentors’ micro-environments were informed by their respective mentoring experience, working style, characteristics, motivations, and clinical, contextual, and personal factors:

*“I think my senior mentors were quite astute and quite experienced, and wise. So, they knew when something was happening in my personal life and helped me...” M2*

*“I think it’s helpful when our mentors do more than just guide us in writing the paper but challenge us to question why we do certain things*.” *M Diary 1*

The host organization’s structure, culture, approach, oversight, and ability to accommodate choice to personalize the mentoring trajectory and experiences shaped the meso-environment:

*“I think, generally, this program has given me a lot of freedom to communicate... and prioritize research.” M3*

*“The PMI allowed talk about other stuff, such as ...medical school, career guidance, advice, even ... personal life...” NP1*

### Sub-domain 3. Macro-environment

The macro-environment encompassed the program’s collaborative environment and the influence of culture, nature, and dynamics of mentoring interactions. The collaborative culture of the PMI promotes open communication and mutual support, which facilitate professional growth:

*“The nature of the work as a doctor is working in a team. So, I think this idea of being able to communicate effectively with your team members and provide constructive feedback will be important in any team-based work. At the same time, part of this culture or spirit of being a doctor is that many times we learn on the job. So, I think the same sort of spirit of giving and teaching are sort of being passed down from the senior. In the same way, I think I see doctors as also having a role of teaching, whether it’s to medical students or to peers”. NP3*

*“It has made me more aware of the importance of research in the field of medicine and how research is a good avenue to give back to the medical community… and create opportunities for medical students to gain exposure and network.” M Diary 4*

**Fig. 1:**
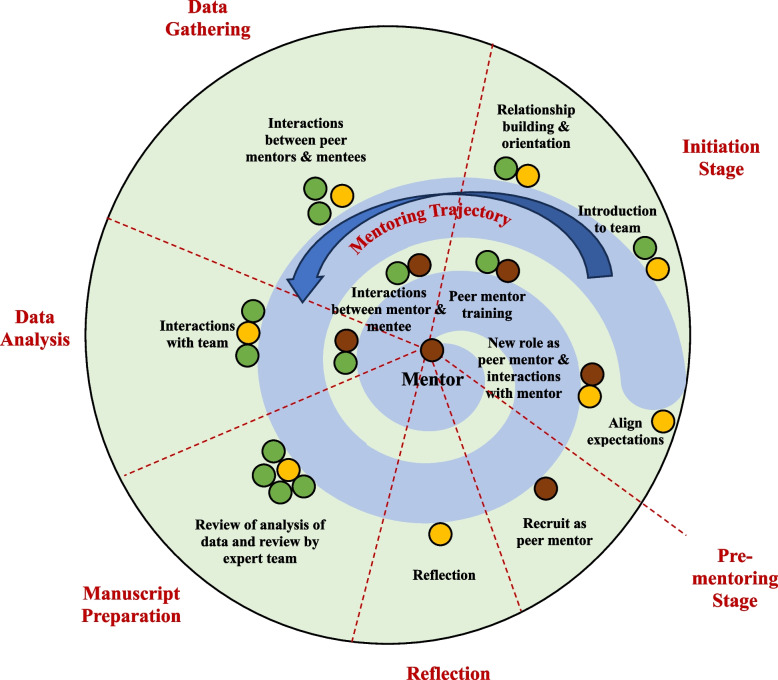
Adapted Mentoring Ecosystem. The green circles symbolize the mentees, light yellow circles reflect peer mentors and dark brown circles represent the mentor

The micro-, meso-, and macro-environments formed the mentoring ecosystem (Fig. 1).

### Domain 2. Professional identity formation

#### Sub-domain 1. Threshold events

Threshold events invite changes in thinking and conduct, as well as enhanced possibilities in future learning. Participants explored how participation in the PMI directly impacted their professional identity:

*“I think research became a significant part of my medical school identity… it provides me with some meaning beyond just academics. So not only am I able to gain research skills, I also feel that I’m playing a part in helping the medical community through research. And getting to know my peers who are in the projects better.” M3*

Mentoring diaries and interviews provided glimpses of the influence of the program on the thinking, decision-making, and conduct of mentees and peer mentors.

*“I feel before being a near-peer, this idea of being genuine, true to who I am, and being supportive and generous in giving... still stayed the same. But I think some of these got more reinforced. Let’s say this idea of being generous or paying it forward or giving back to people got reinforced during my process as a near-peer.” NP3*

*“I think everyone has a certain level of communication skills. And I think that the process of me leading the team helped to strengthen that.” NP4*

### Sub-domain 2. Identity work

The participants realized that, at times, adaptations were necessary to maintain their overall identity. NP2, for instance, recalled how they adapted their working style to uphold the identity of a competent peer mentor:

*“I did feel I had to alter my working style at times. It was about striking a balance. At times, you may need to take a harder approach to get them to respond or turn up work in time. Because if you are too lax, they may keep pushing deadlines, but at the same time, there’s also a balance in terms of not being too harsh on people, and also giving enough time for them to discover things for themselves.” NP2*

With greater experience, access to longitudinal mentoring support, and growing knowledge of the mentoring process, environment, and relationships, participants reported greater confidence and competence, which often extended beyond their role in the PMI.

*“Although I sometimes still find myself a bit lost, or having some struggles, I feel that I’m capable of finding a way for myself to get through them. To have more confidence, persevere, and tell myself that I’m able to do well eventually.” M3*

*“Initially, I think when I was a mentee, I was just more focused on my own development. Subsequently, when I see from the eyes… of a near-peer, I think the idea was also to see how I can support other people in their development, how I can be more sensitive to what their needs [are] and provide advice…. So I think the perspective changed from focusing on myself to focusing on others.” NP3*

The participants recognized how their involvement in the PMI helped to shape their professional identities. NP2 and NP10 described how interactions with their mentor influenced their perceptions of an ideal physician:

*“The influence from a mentor has changed the way I look at certain specialties and changed my outlook on life. It has made me more focused on being less materialistic and more on fields of work where I can provide a listening ear to people, and generally provide more holistic care because that’s what my mentor did for me.” NP2*

*“I think one of the greater positive things that my mentors in the PMI have impressed upon me is this amazing sense of patience and really caring about what others are going through.” NP10*

### Sub-domain 3. Reflection

Peer mentors reflected as they deliberated on their options more than their less experienced mentees. These reflections assumed two forms: reflection-in-action [60] and reflection-on-action [61]. Reflection-in-action informed advanced decisions on adaptations to the belief system:

*“I picked up the importance of how to set goals effectively, not just by seeing my mentors do it, but also through the research that I’m involved with, and seeing the effects and importance of it right from the beginning.” NP1*

*“The one point that is immediately relevant to me now is how my research on professional identity formation (PIF) has made me very acutely aware of how I am being influenced during my clinical postings. I am now able to put a name to what I am experiencing or relate it to a theory.” NP Diary 5*

Conversely, when critical events were not recognized at the moment or when responses were felt to be inadequate, peer mentors and mentees reveal reflection-on-action or reflection after the fact. This practice was especially evident amongst mentees. Lessons learned influenced thinking and planning and informed later practice, as described in the following accounts:

*“When we first started, we did not always ask for clarifications, because we thought that this [was] something that we should know already. We tried to figure it out ourselves but by the time we tried to… we realized that none of us [knew how to] do it and we were already in too deep.” M4*

*“When I face very difficult or bad experiences where there are ethical issues, or when I get scolded or receive criticism, I will always reflect on how I can go about changing and going further to become better...” NP3*

### Sub-domain 4. Splinting and patching

Mentees and peer mentors also unveiled instances of identity work through splinting and patching*.* A less experienced mentee discusses an example of splinting:

*“If there are other more experienced people on the team, I will tend to take a step back and just listen to and follow instructions.” M4*

Meanwhile, NP1’s refinement of their mentoring approach based on role modeling provided an example of patching*:*

“*From mentoring and leadership experiences, I realized that I do not see myself as someone who tells people what to do. That affected my mentorship or my ability to lead… So what I learned [was] the need to demand a certain sort of command over people, especially when you are leading a project because, without a proper hierarchy, or a proper flow or chain of command, it does affect… the process.*” *NP1.*

## Discussion

This study explores the multiple converging factors that support the development of mentees’ and peer mentors’ professional identities as they navigate stages of the mentoring trajectory within the mentoring ecosystem of a peer mentoring program. This professional development is fostered through the interaction of individual, group, and system-level facets. We have previously described PIF within the PMI, focusing on the individual experiences of the mentees and peer mentors [11]. Here, the multiple levels of the ecological systems framework help to advance our understanding of the complex factors that contribute to the evolution of PIF [9]. The theory has been recently used to better understand interactions in health professions education. Hamwey et al. used the model to illustrate the numerous factors that impact the academic performance of health professions learners [62]. Bluteau and colleagues demonstrated that students in a longitudinal interprofessional education program are better able to consider the factors that affect healthcare from individual (micro) to group (meso) and, ultimately, to higher organizational (exo) and cultural (macro) levels as they progressed through their training [63].

Examining complexities in an interrelated manner is a strength of systems theories [9]. Accordingly, our study highlighted that the components of the mentoring ecosystem are dynamic and interconnected. Contextual factors, such as a participant’s personal characteristics or motivation, play an important role in shaping mentoring relationships and outcomes and can both enable and constrain mentoring and identity formation. The participants acknowledged the role of stakeholders, including the PMI program itself, in providing resources, opportunities, and a support network/community within the mentoring ecosystem. Exposure to the PMI’s belief system and culture also impacts the mentoring experience, progress, and outcomes.

This adapted mentoring ecosystem also considers the mentee’s reinvolvement in the PMI as a peer mentor, where they revisit the same mentoring stages, albeit in different roles that accord more responsibilities. This extended mentoring trajectory toward the more central role in the PMI that mentors usually play provides a better appreciation of the impact of interactions between stakeholders and the effects of reflection on new life experiences upon individual belief systems as professional identity is formed. It also provides further depth to our appreciation of mentoring processes.

Notably, the trajectory within the mentoring ecosystem is not rigid. Rather, it acknowledges variations in the belief systems, narratives, contextual considerations, developing competencies, and environmental and contextual considerations of each member. It also impacts and is impacted by the mentoring structure, culture, organization, and the healthcare and education system in which it exists. To guide this personalized, longitudinal, and holistic support, the process relies on stage-based and competency-based assessments that can direct trained mentors and peer mentors, as well as a mentoring structure that limits external influences upon this process and confines responses within program expectations and codes of practice. This fosters greater confidence and the ability to ‘think on your feet’ or practice reflection-in-action [9–12]. With more experience, mentees and peer mentors move towards adopting and trialing the desired characteristics role modeled by senior mentors (*patching*) [45]. Perhaps just as importantly, the theory highlights how reflection-in-action and identity work both influence, and are influenced by, the mentee’s ability to analyze, reflect upon, and learn from interactions within the meso-environment and the macro-environment. The mentee’s ability ultimately shapes the developing self-concepts of personhood and identity.

The insights provided suggest similarities with Communities of Practice (CoP), “a persistent, sustaining social network of individuals who share and develop an overlapping knowledge base, set of beliefs, values and history and experiences focused on a common practice and/or enterprise” [8]. This notion is underscored by the presence of the mentoring ecosystem’s structured approach and mentoring trajectory that moves participants from legitimate peripheral participation to a central role at the heart of the program (Fig. 1). This movement is fostered by longitudinal and personalized mentoring support and a nurturing mentoring environment. Imagining the mentoring ecosystem as a CoP, a key element in nurturing PIF, allows a deeper understanding of mentoring’s impact on PIF.

Our findings have educational implications. The ecological systems theory underscores the importance of considering the interaction of various factors and stakeholders when designing a mentoring program, including ensuring mentor support, comprehensive peer mentor training, and a nurturing organizational culture. This holistic approach acknowledges the interconnectedness of the mentoring ecosystem, where each component, from individual relationships to broader cultural influences, plays a vital role in supporting PIF.

### Limitations

The use of single time-point interviews and retrospective accounts as the primary source of data in this study is susceptible to recall and social desirability biases [64]. This is suggested by the predominantly positive mentoring narratives, with little discussion of the known hierarchal and unequal power distributions inherent to many mentoring relationships [65]. Further, although professional identity formation occurs within the context of cultural and societal norms and expectations, particularly within a multi-ethnic and multi-cultural country like Singapore, we did not explore the impact of the mentees’ or peer mentors’ gender or socio-cultural backgrounds on their experiences within the PMI or on their mentoring interactions. There are also limitations due to the small sample size and the depth of the data collected.

## Conclusion

In this study of mentorship within the PMI, the mentoring ecosystem model underscores the nuances and complexities of mentorship and provides a framework for PIF. A deeper understanding of the multiple factors that converge to facilitate the professional development of mentees can help educators develop and implement structured peer mentorship programs that better support reflective practice and identity work.

## Data Availability

The dataset(s) supporting the conclusions of this article is(are) included within the article (and its additional file(s)).

## Abbreviations

PIF: Professional identity formation
PMI: Palliative medicine initiative
NCCS: National cancer centre Singapore
SEBA: Systematic evidence-based approach
CoP: Community of practice
